# Inside the hypoxic tumour: reprogramming of the DDR and radioresistance

**DOI:** 10.1038/s41420-020-00311-0

**Published:** 2020-08-18

**Authors:** Katheryn Begg, Mahvash Tavassoli

**Affiliations:** Head and Neck Oncology Group, Centre for Host Microbiome Interaction, King’s College London, Hodgkin Building, London, SE1 1UL UK

**Keywords:** Radiotherapy, Cancer

## Abstract

The hypoxic tumour is a chaotic landscape of struggle and adaption. Against the adversity of oxygen starvation, hypoxic cancer cells initiate a reprogramming of transcriptional activities, allowing for survival, metastasis and treatment failure. This makes hypoxia a crucial feature of aggressive tumours. Its importance, to cancer and other diseases, was recognised by the award of the 2019 Nobel Prize in Physiology or Medicine for research contributing to our understanding of the cellular response to oxygen deprivation. For cancers with limited treatment options, for example those that rely heavily on radiotherapy, the results of hypoxic adaption are particularly restrictive to treatment success. A fundamental aspect of this hypoxic reprogramming with direct relevance to radioresistance, is the alteration to the DNA damage response, a complex set of intermingling processes that guide the cell (for good or for bad) towards DNA repair or cell death. These alterations, compounded by the fact that oxygen is required to induce damage to DNA during radiotherapy, means that hypoxia represents a persistent obstacle in the treatment of many solid tumours. Considerable research has been done to reverse, correct or diminish hypoxia’s power over successful treatment. Though many clinical trials have been performed or are ongoing, particularly in the context of imaging studies and biomarker discovery, this research has yet to inform clinical practice. Indeed, the only hypoxia intervention incorporated into standard of care is the use of the hypoxia-activated prodrug Nimorazole, for head and neck cancer patients in Denmark. Decades of research have allowed us to build a picture of the shift in the DNA repair capabilities of hypoxic cancer cells. A literature consensus tells us that key signal transducers of this response are upregulated, where repair proteins are downregulated. However, a complete understanding of how these alterations lead to radioresistance is yet to come.

## Facts

- Hypoxia is present in almost every solid tumour
- Hypoxia is a major barrier to effective radiotherapy and is associated with radioresistance
- The hypoxic tumour is highly heterogenous, with regions of chronic and acute hypoxia, altered pH and immune infiltration
- Differences in gene expression and protein function can occur between acute or chronic, and mild or severe hypoxia
- All DNA damage response (DDR) pathways, including homologous recombination, non-homologous end joining, miss-match repair and the Fanconi anaemia pathways have been shown to suffer alterations in hypoxia
- Activation of DDR transducer protein ATM is seen in severe hypoxia, in the absence of classical ATM-activating features such as double strand DNA breaks
- ATR is also activated, most likely in response to hypoxia-induced replication stress
- However, downregulation of DNA repair effector proteins such as RAD51 and BRCA1/2 is seen
- Results of DDR reprogramming include genetic instability, aberrant cell cycle and apoptotic control

## Open questions

- Precisely how do alterations to the DDR in hypoxia lead to radioresistance? For example, when genomic instability and generation of radioresistant clones takes several cell divisions to set in, how does a decrease in DNA repair ability lead to increased radioresistance?
- What aspects of the hypoxic response could be targeted to radiosensitise or more effectively treat tumours, particularly in the context of DDR? For example, can we target upregulated DDR transducers such as ATM, ATR and DNA-PKcs or induce synthetic lethality following downregulation of DNA repair effectors?
- Do different types of cancers have different patterns of DDR alteration within hypoxic tumours? This particularly needs further research as tissues have been shown to have different oxygen pressures, levels of hypoxia and hypoxic heterogeneity.
- Can we use the data on this subject to develop a biomarker signature of hypoxia-induced radioresistance, as we have done using hypoxia as a single parameter?
- How can we monitor hypoxic tumours during the course of a patient’s disease to help guide treatment?
- How can we ensure reliable reporting and interpretation of in vitro and in vivo data?

## Introduction

Hypoxia is present in almost every solid tumour, an inevitability of cancer’s characteristic disorganised and functionally inefficient vasculature, rapid growth and demanding metabolism^1^. The result is a comprehensive re-writing of transcriptional programs, up/downregulating certain genes and proteins allowing cells to evade apoptosis and migrate to areas with better oxygen perfusion. Crucially, this microenvironmentally induced intracellular shift also results in genomic instability (GI), alterations to DNA repair and resistance to cell killing by cancer therapies. After decades of research, it has become clear that the relevance of hypoxia for both oncogenesis and treatment resistance is inescapable.

In the context of radiotherapy (RT), a link between resistance and low intratumoral oxygen pressure has been known since the publication of a study modelling oxygen flow in lung tumours 65 years ago^2^. For some cancers, such as Head and Neck Squamous cell carcinomas (HNSCCs) hypoxia is a major contributing factor to local RT failure^3–6^. Advances in developing technologies allowing for more precise delivery of RT, imaging of tumours and sensitisation to treatment while protecting normal tissues, have led to improved locoregional control and quality of life for patients^7,8^. However, since treatment for many cancers like HNSCCs (which incidentally are also some of the most hypoxic), depends on RT the hypoxic problem remains particularly pertinent^9^.

In recent years efforts have been made to correct or reverse hypoxia, including administering hyperbaric oxygen therapy to patients^10,11^, reducing cellular oxygen consumption^12^ and increasing blood vessel functionality^13,14^. To more precisely tackle resistance induced by hypoxia researchers have also sought to lower the threshold of treatability of hypoxic tumours by use of sensitizers^15–17^. As a third arm in our battle plan, research has also gone into developing methods to detect hypoxia, including the use of specialised imaging techniques (PET/CT scanning combined with hypoxia-detecting radionuclides) often studied in tandem with genetic signatures seeking to genotypically define these tumours^18,19^.

However, very few of these advancements have allowed us to overcome hypoxia-induced radioresistance (RR). Though research activity in this area remains strong, a more complete understanding of how the hypoxic environment contributes to RR, particularly by modulation of potentially targetable DNA damage response (DDR) pathways, is warranted. This review will outline our current knowledge of the molecular processes that underpin hypoxic RR particularly in the reprogramming of the DDR.

## Radiotherapy mechanism of action—the requirement of oxygen

Seminal work by Gray and colleagues during the 1950s proved that the efficacy of RT was dependent on the availability of oxygen within the tissue^2,20,21^. Radiation induces damage through the direct and indirect generation of double stranded breaks (DSBs) in DNA. In the presence of oxygen, damage induced is 2.5–3 times more likely to end in cell death^22^. This effect is best explained by the Oxygen Fixation Hypothesis, where radicals produced directly or indirectly by ionizing radiation (IR) are oxidised to DNA in the presence of oxygen^23^, making the damage irreversible^24^. This last point is crucial to the hypothesis, with the notion that these lesions cannot be restored to an undamaged state as the damage is “fixed” to DNA by oxidisation^25^. Thus, without oxygen, damage induced is transient and hypoxic cells experience far reduced radiation-associated damage.

Though crucial, the requirement of oxygen to induce damage is not where the story ends for RR, as it does not fully explain the level of RR we observe. This is evidenced by the fact that restoration of oxygen to tumours (for example through applying hyperbaric oxygen) does not restore radiosensitivity^26^. Importantly, it also does not account for changes that occur with respect to DNA repair, which have been shown to be crucial in impacting the radiation response^27^, as these changes are retained past the point of radiotherapy administration.

## The landscape of the hypoxic tumour

**Fig. 1:**
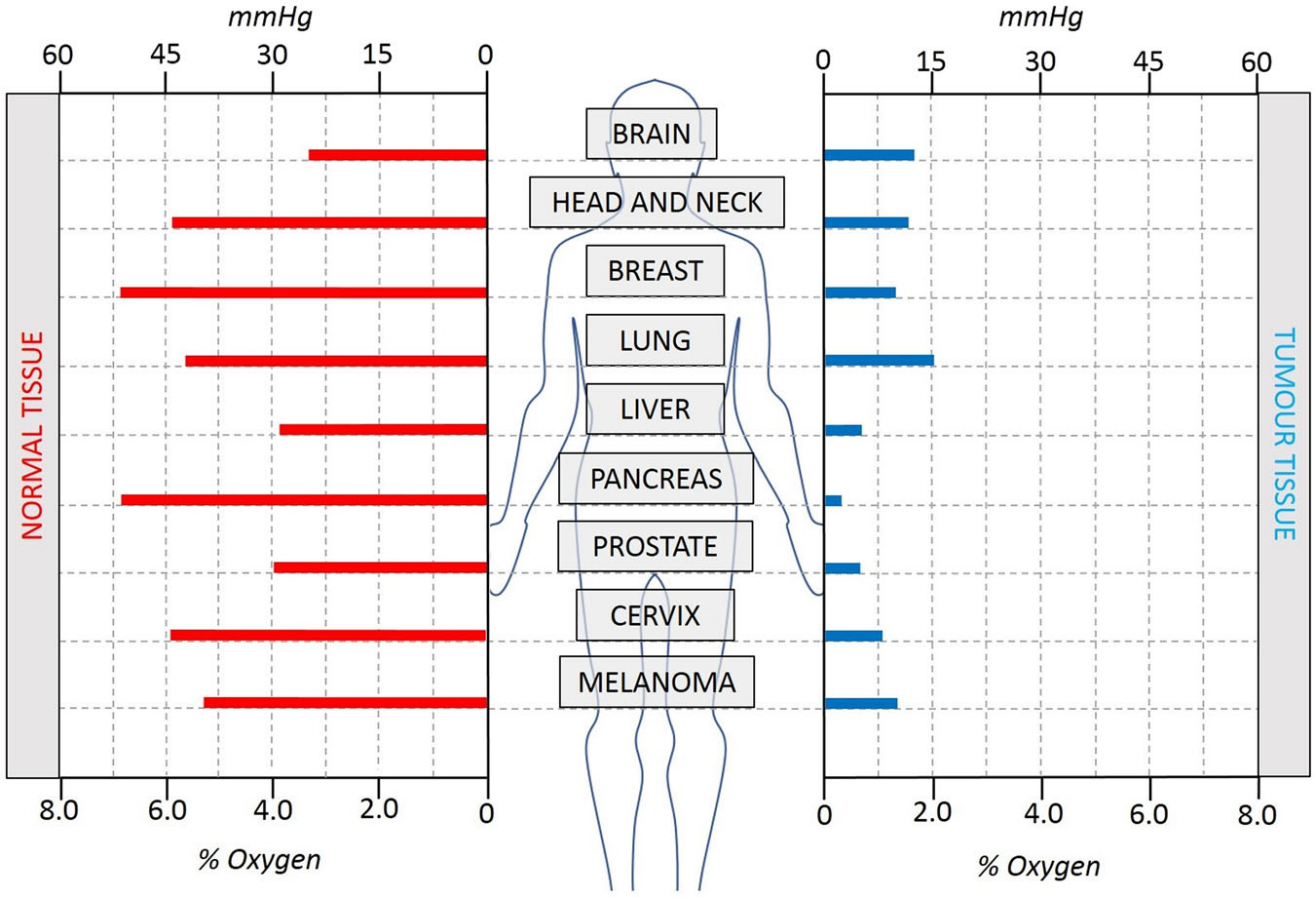
Approximate oxygen levels reported in different tissues in mmHg (used in in vivo experiments) and % O_2_, (used in in vitro experiments). Note that normal tissue normoxia (or physoxia) is considerably less than the 21% O_2_ used in vitro as normoxia. Adapted from McKeown^139^, Liu^140^ and Graham^26^.

In vivo, the hypoxic region exists on a gradient of oxygen pressures, with oxygen levels throughout the tissue ranging between <0.5% (severe hypoxia), 0.5–3% (mild hypoxia) and 0% (anoxia) with around 6% considered physoxia (see Table 1 for definitions). Tissue oxygen pressures are usually measured in mmHg. However, since the majority of research on hypoxia and the DDR has been performed in vitro, where oxygen levels are measured in percent, for the purpose of this review % O_2_ will be referred to predominantly. The difference between in vivo and in vitro measurements of oxygen is important though, with tissue normoxia (physoxia) classified at around 6% O_2_ or 30 mmHg, and in vitro normoxia being around 21% O_2_ (Fig. 1).

**Table 1:** Glossary of terms. Multiple classifications of the terms used to describe hypoxia exist throughout the literature. This represents a general consensus and what is used in this review.

Term	Definition
Hypoxia	Reduced oxygen levels, usually ≤1% O_2_ (~5 mmHg) in in vitro studies
Normoxia	Normal atmospheric oxygen used in in vitro studies, 21% O_2_ (~160 mgHg)
Physoxia	Physiological levels of oxygen in tissues, between 3-7% (~20-50 mmHg), tissue specific (see Fig. 1)
Anoxia	Complete absence of oxygen (0%)
Severe hypoxia	<0.5% O_2_
Mild hypoxia	>5% O_2_–3% O_2_
Acute hypoxia	Incubation in hypoxic conditions <18–24 h
Chronic hypoxia	Incubation in hypoxic conditions >24 h
Radiobiological hypoxia	Oxygen levels where the efficacy of radiotherapy is half maximal, ~3 mmHg/0.4% O_2_

**Fig. 2:**
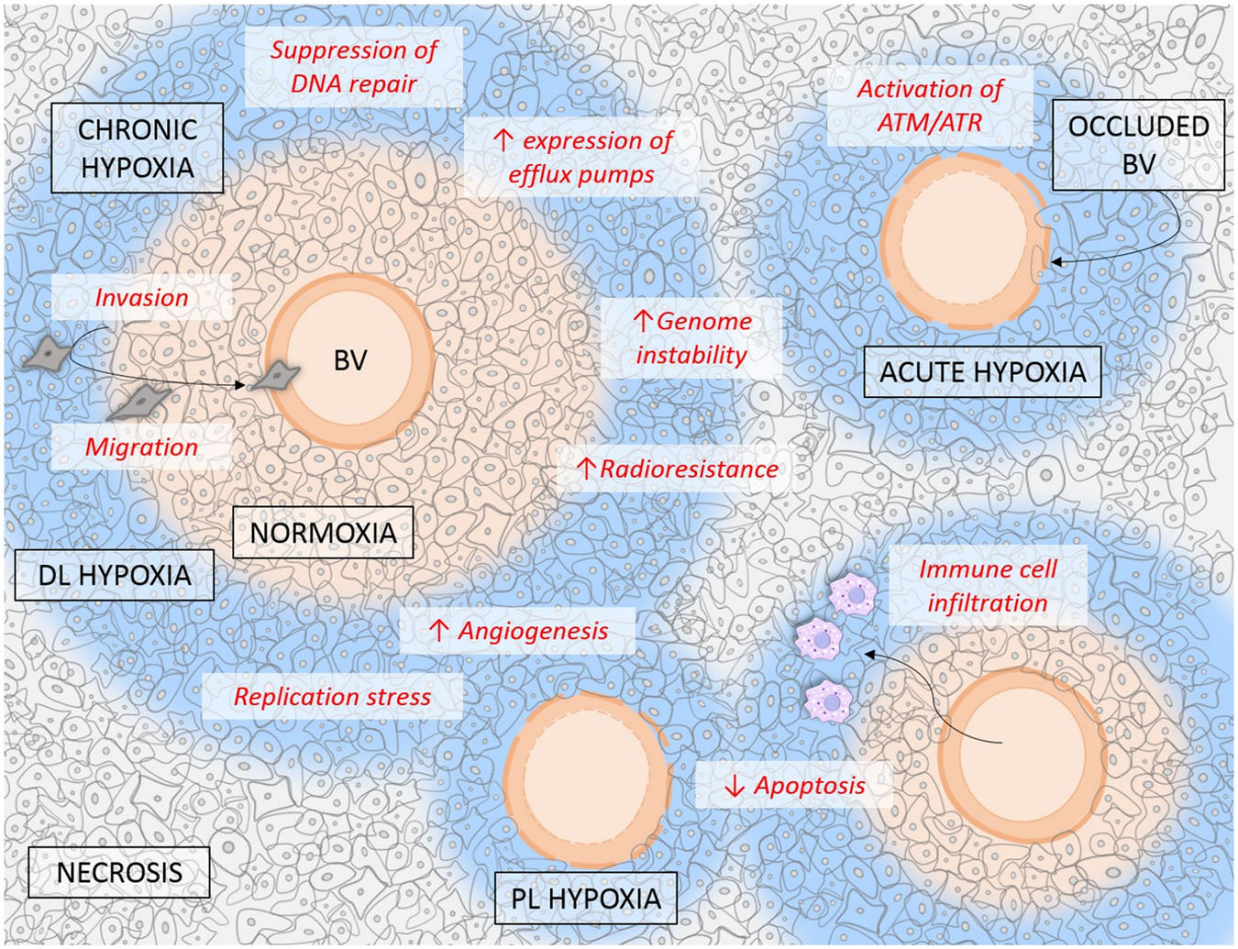
The heterogeneity of the hypoxic tumour. Tumours suffer from reduced oxygen availability due to the disorganised nature of the vasculature. Where occlusion of a blood vessel (BV) occurs, tumours are said to be under perfusion limited hypoxia (PL hypoxia). Where lack of oxygen is a function of distance from the vessel, cells experience diffusion limited (DL) hypoxia. When these states are temporary (<24 h) it is said to be acute or chronic when >24 h. Within hypoxia tumour cells undergo considerable genetic reprogramming, contributing to therapy resistance and metastatic behaviour.

The hypoxic tumour is a space of restricted proliferation (particularly when oxygen levels are <0.5% O_2_), cell cycle arrest and decreased protein synthesis juxtaposed against accelerated aggressivity, microenvironmental interactions and altered pH^28–30^. At the most oxygen-depleted border exists the barren land of necrosis, with the highly proliferating and comparatively treatment-sensitive aerobic cells closest to the blood vessel (Fig. 2).

Tumour hypoxia does not develop in a linear fashion and is highly heterogenous and changeable. The hypoxic tumour is dynamic, with fluctuating vessel functionality and cycling oxygen levels creating regions of acute and chronic hypoxia^31^. Where part of the tissue may suffer acute hypoxia after temporary occlusion of a blood vessel (so-called perfusion limited in which oxygen-deprivation cycles last sometimes minutes, sometimes hours before subsequent reoxygenation), chronic hypoxia is diffusion limited, where oxygen levels become a factor of distance from the blood vessel^31^. Compounded with this is the differing rates of oxygen consumption and responsiveness to oxygen availability in cells within the tissue. To be able to fully understand and ultimately treat the hypoxic tumour, it must be remembered that the changeability of oxygen concentrations in the hypoxic tumour also, predictably, influences its behaviour and response to treatment. In the context of radiotherapy, cells with O_2_ levels <1% is where we see most resistance, so called radiobiological hypoxia^24^. Whether this is acute (and therefore followed by reoxygenation) or chronic (oxygen deprived for more than 24 h) can have marked differences on the ensuing genomic and proteomic changes that ultimately allow for hypoxic survival^32^. Thus, within one tumour, different regions are likely to have a completely different response to the same dose of radiotherapy.

Aside from the oxygen status, the involvement of other environmental features affected by hypoxia must be considered. An additional outcome of hypoxic adaption is the concomitant phenotypic shift of the microenvironment. It is now accepted that hypoxia can induce inflammation^33^, demonstrated even by patients who develop mountain sickness after prolonged periods at high altitude^34^. Hypoxic tumours are known to have higher infiltration of pro-tumour immune cells such as M2 macrophages^35^, a feature known to be involved in RR^36,37^. The same could also be said with respect to the hypoxic-induction of highly RT resistant cancer stem cells^38^. Though this subject needs further research, the likelihood of an interplay between intracellular genetic reprogramming as a result of hypoxic adaption and the microenvironment in mediating radioresponse is strong.

## Genetic reprogramming—the HIFs

Within this chaotic showground of heterogeneity, and at the core of cellular adaption to hypoxia are the altered genetic pathways that push for survival against adversity. Commonly dysregulated genes include GLUT1 (involved in altered glucose metabolism), VEGF (involved in neoangiogenesis) and LOX (involved in remodelling of the extracellular matrix)^7^.

In 2019, the Nobel Prize in Physiology or Medicine was awarded to three scientists, Gregg Semenza, William Kaelin and Sir Peter Ratcliffe, for their contributions to our understanding of cellular oxygen-sensing mechanisms^39^. This included the discovery of a group of transcription factors regulated by hypoxia that allow for cellular adaption^40^. These hypoxia-inducible factor (HIF) proteins (HIF-1-3) are transcription factors composed of two subunits. The α subunits, reside in the cytoplasm and are subject to rapid degradation (5–10 min^41^) under normal circumstances. This degradation is mediated by the actions of Prolyl-hydroxylases (PHD1-4) which hydroxylate HIF-α at the oxygen-dependent-degradation domains (ODDD). Of note PHD2 and PHD3 are themselves transcriptional targets of HIF, alluding to possible negative feedback systems in place, though conflicting results suggest this system doesn’t always function effectively to constrain cancer growth^42–44^. Subsequently, hydroxylation by PHDs recruits the Von-Hippel-Landau (VHL) protein^45^. This, alongside other proteins, forms an E3 ubiquitin ligase complex ubiquitinating HIF-α for proteasomal degradation.

In hypoxia, due to the lack of molecular oxygen needed for hydroxylation^42,46^, this degradation cascade does not take place, and HIF-α subunits translocate to the nucleus to associate with HIF-β subunits^47^. The HIF complex in interaction with its coactivators p300 and Creb-binding protein (CBP), then binds to hypoxia response elements (HREs) in DNA to initiate transcription of HIF-target genes.

Additional layers of HIF regulation also exist to keep this pathway in check, such as factor inhibiting HIF (FIH) which hydroxylates HIF subunits at asparagine residues, blocking their association with p300/CBP^48^. Some evidence has shown that HIFs also undergo other posttranslational modifications including phosphorylation and acetylation as a further method of regulation^42,47^. However, as with many such processes in cancer, it can be aberrantly controlled, including in the hypoxia-independent stabilisation of HIF-α by oncogenes such as EGFR and mTOR^49,50^, and depletion of HIF-regulatory factors^42,51^. The HIF proteins themselves can interact with a number of factors relevant in cancer, such as p53 mutants present in human papillomavirus (HPV)-negative HNSCCs and non-small cell lung cancers (NSCLCs), resulting in transcriptional control of pro-tumorigenic genes^52^. Since both HIFs and p53 compete for binding of p300/CBP to enact transcriptional control, the HIFs have a unique relationship with this highly cancer-relevant protein^53^. Inactivation of p53’s transcriptional abilities has been observed^54^ though again, conflicting results exist for this^55^.

Most of the work investigating HIF-directed transcriptional changes in hypoxia has focussed on the actions of the best-known HIF, HIF-1. However, both HIF-2 and HIF-3 also play a role in hypoxic transcriptional control^42^. Interestingly, relative expression of the HIFs has been shown to differ between hypoxic tissues, demonstrating that each may have specific functions^56^. In some cases they may indeed work in concert, as HIF-2 has been shown to be induced when HIF-1 is depleted^50^.

HIFs are master regulators of the hypoxic response. And concurrent with the notion that hypoxic tumours are radioresistant, depletion of HIF-1α in tumour models radiosensitises cells^41^. One study showed that intermittent hypoxia showed less radiation-induced cell death both in vitro and in mice via stabilisation of HIF-1α^57^. This investigation also found that intermittent hypoxia had a more significant effect than chronic hypoxia. HIF-1α has also been shown to function via the HIF-1α–Myc pathway, in which HIF-1α competes with the transcription activator Myc for Sp1 binding in the target gene promoter, to downregulate mismatch repair (MMR) genes MSH2 and MSH6 in 1% O_2_^58,59^.

However, how exactly HIFs contribute to RR of the hypoxic tumour, be it through their transcriptional functions or interactions with other proteins, is so far unresolved. Notably, some radio- and chemotherapies themselves upregulate or stabilise HIFs^41^.

Though genetic reprogramming in hypoxia can lead to a number of alterations, for the purpose of this review, we will focus on those associated with RR and DDR. For more general reviews see Schito^60^ and Tsai^61^.

## Reprogramming of the DDR

The DDR is a complex process consisting of overlapping and interconnected pathways initiated by different forms of DNA damage. Arguably one of the most important homeostatic processes, it allows us to withstand constant and numerous DNA damage-inducing insults. The result of this protection ensures that only reliable genomes are passed on to the next cellular generation. For cancer, considering both the power of mutagenesis in driving oncogenic potential, and the fact that many cancer therapies function by inducing DNA damage, the DDR has considerable relevance for therapy resistance and tumour progression.

Repair of DNA is a tale of three acts: firstly, the damage propagates a signal that recruits *sensors* to the site of damage; secondly the signal is amplified by *transducers*; and thirdly, response pathways are initiated by *effectors*. For each part of the process, well-defined (though not exclusive) sets of proteins act as sensors, transducers and effectors respectively^28^. Shrouding these repair processes are signals to stall the cell cycle (initiated by Chk1- or Chk2-activated CDC25 and p21) to allow time for clearance of this damage, and initiation of apoptotic pathways (for example as initiated by ATM’s interaction with p53) if the repair is unsuccessful^62,63^.

For the repair of radiation-induced DSBs, two primary pathways are put to use, homologous recombination (HR) and non-homologous end joining (NHEJ). The former, considered less error-prone, uses sister chromatids to repair DNA and as such can only take place during G2/S phase of the cell cycle. NHEJ predominates in G1 but can occur at any stage of the cell cycle and often results in the generation of insertion/deletion mutations, which have the potential to lead to more oncogenic alterations. HR is mediated primarily by the recruitment to sites of damage of master transducer of the DSB response, ATM (Ataxia Telangiectasia mutated, a phosphoinositide-3-kinase-related protein kinase (PIKK)) following detection by the MRN complex (composed of MRE11-RAD50-NBS1), which initiates activity of effectors including RAD51 and BRCA1. NHEJ occurs following sensing of damage by the Ku proteins, Ku70 and Ku80, and signal transduction of Ku in complex with DNA-PKcs (DNA-dependent protein kinase catalytic subunit—together forming DNA-PK) and subsequent activity of effectors DNA Ligase IV (LIGIV) and XRCC4^64^.

Alteration of DDR pathways has been seen across many cancers compared to normal tissue. Perhaps the most well-known are the mutations in BRCA1/2 in aggressive hereditary breast and ovarian cancers^65^. Understandably, where hypoxia represents an exaggerated form of aggressive tumours, the DDR pathways in hypoxia operate differently to those in normoxia. Indeed, this is true for every aspect of the DDR process. DNA damage in the form of DSBs is reduced in conditions of hypoxia <1% O_2_, and hypoxia alone does not induce DSBs^24,66^. Research has shown that different members of the DDR pathways can be either activated or downregulated in conditions of low oxygen (see Tables 2, 3 and 4 for reported alterations to HR, NHEJ and mismatch repair (MMR) pathways). Crucially, whether the cells are in acute or chronic hypoxia (< or >24 h) and at what level of oxygen depletion, may define the ensuing response. Despite this delineation, there lacks within the literature proper reporting of experiments carried out in either acute or chronic, mild or severe hypoxia, with interchangeability in use of terms. See Table 1 for a consensus of parameters used with these definitions.

**Table 2:** A non-exhaustive list showing alterations to sensors, transducers and effectors of the homologours recombination (HR) pathways in hypoxia.

Protein	Role in DDR	Mechanism of alteration	Alteration, conditions and consequences	Reference
NBS1	Sensor of DSBs in HR, activated ATM as part of the MRN complex	• Pas-B domain of HIF-1-α	• Downregulated in chronic mild hypoxia (>5 days, 1% O_2_) • Downregulation in acute mild (16 h, 1% O_2_) • Resulted in induction of γH2AX and 53BP1 foci	Cowman^69^ To^59^
MRE11	Sensor of DSBs in HR, activated ATM as part of the MRN complex	• ?	• Downregulated in chronic mild hypoxia (>5 days, 1% O_2_)	Cowman^69^
ATM	Transducer of HR in DSB repair	• Autophosphorylation at Ser1981	• Activated in acute hypoxia (<0.02% O_2_) • Increased expression and activity (<0.05% O_2_, 12–24 h) • Mediated by Src and AMPK signalling	Hashimoto^88^ Bencokova^28^
ATR	Transducer of DNA repair, induced by replication stress	• ?	• Activated in acute (<0.2% O_2_) • Resulted in phosphorylated p53 and accumulation and growth arrest	Hammond^75^
RAD51	Effector of DSB repair in HR	• E2F4/P130 • LSD1 • EZH2	• Downregulation in chronic, severe hypoxia, (0.2% O_2_, 48–72 h, and 0.01% or 0.5%, 24–48 h) • Decreased radioresistance • Increased genomic instability • Downregulation in 2% O2 > 6 h	Meng^119^ Bindra 2006/4^111^ Oliveira^82^
RAD52	Effector of DSB repair in HR	• miR-210 • miR-373 • miR-210	• Decreased mRNA expression (0.2% O_2_, 48–72 h) • Downregulated (0.1% O_2_, 24 h)	Meng^119^ Crosby^118^
RAD54	Motor protein, effector of DSB repair in HR	• ?	• Decreased mRNA expression (0.2% O_2_, 48–72 h)	Meng^119^
BRCA1	Effector of DSB repair in HR	• E2F4/P130 • H3K4 demethylation via LSD1	• Downregulation in chronic, severe hypoxia (0.01% O_2_, 48 h) • Decreased mRNA expression (0.2% O_2_, 48–72 h) • Downregulation in 2% O2 > 6 h • Decreased radioresistance	Meng^119^ Lu^117^ Bindra^120^ Oliveira^82^
BRCA2	Effector of DSB repair in HR	• ?	• Decreased expression (0.2% O_2_, 48–72 h)	Meng^119^

**Table 3:** A non-exhaustive list showing alterations to sensors, transducers and effectors of the non-homologous end joining (NHEJ) pathways in hypoxia.

Protein	Role in DDR	Mechanism of alteration	Alteration, conditions and consequences	Reference
Ku70	Sensor in NHEJ pathways, recruits DNA-PKcs In complex with Ku80	• ?	• Decreased mRNA expression (0.2% O_2_, 48–72 h) • Upregulation (<0.1% O_2_, > 36 h) • Downregulation in cervical tumour sections • Upregulation (1% O_2_, 2–8 h)	Meng^119^ Ren^83^ Lara^81^ Um^84^
Ku80	Sensor in NHEJ pathways, recruits DNA-PKcs In complex with Ku70	• ?	• Upregulation (<0.1% O_2_, > 36 h) • Downregulation (2% O_2_, > 6 h) • Downregulation in cervical tumour sections • Upregulation (1% O_2_, 2–8 h)	Oliveira^82^ Ren^83^ Lara^81^ Um^84^
DNA-PKcs	Transducer of NHEJ pathway	• Autophosphorylation at Ser2056	• Decreased mRNA expression (0.2% O_2_, 48–72 h) • Increased expression and activity (<0.05% O_2_, 12–24 h) • Activated in mild hypoxia (0.1–1% O_2_) led to positive regulation of HIF-1 and upregulation of GLUT1	Meng^119^ Hashimoto^88^ Bouquet^103^
DNA LIGIV	Effector of NHEJ repair	• ?	• Decreased mRNA expression (0.2% O_2_, 48–72 h)	Meng^119^
Xrcc4	Effector of NHEJ repair	• ?	• Decreased mRNA expression (0.2% O_2_, 48–72 h)	Meng^119^

**Table 4:** A non-exhaustive list showing alterations to sensors, transducers and effectors of the mismatch repair pathway in hypoxia.

Protein	Role in DDR	Mechanism of alteration	Alteration, conditions and consequences	Reference
MLH1	Dimerises to PMS2 to form the MutLα complex in MMR	• Mad1/Max • Mnt/Max • DEC1/2 • miR-155 • LSD1 • HDAC • Hypoacetylation/hypermethylation on H3	• Downregulation (24–48 h, 1% O_2_) • Downregulation in (48 h, 0.01% O_2_) • Increased expression (3–48 h, 1% O_2_) resulting in genomic instability in stem cells	Bindra^121,127^ Mihaylova^128^ Nakamura^129^ Rodriguez-Jimenez^145^ Lu^115^
PMS2	Dimerises to MLH1 to form the MutLα complex in MMR	• ?	• Downregulation at protein level (24–48 h, 1% O_2_) • Resulting in genomic instability in stem cells	Mihaylova^128^ Rodriguez-Jimenez^145^
MSH2	Dimerises with MSH6 forms the MutSα complex in MMR	• Myc/Max • HIF1-α via Sp1 • miR-155 • H • P53	• Downregulation (16–48 h, 1% O_2_)	Bindra^121,127^ Koshiji^58^
MSH6	Dimerises with MSH6 forms the MutSα complex in MMR	• HIF1-α via Sp1 • miR-155 • HDAC • P53 • Hypoacetylation/hypermethylation on H3	• Downregulation (16–48 h, 1% O_2_) • Increased expression (3–48 h, 1% O_2_) resulting in genomic instability in stem cells	Koshiji^58^ Rodriguez-Jimenez^145^

### Sensors

**Fig. 3:**
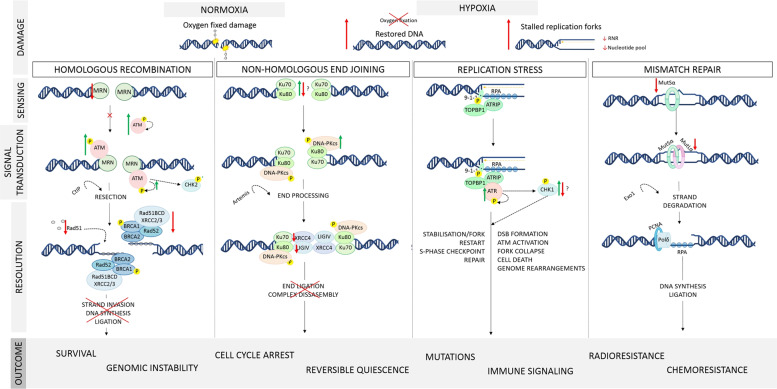
Hypoxia induces changes to a number of proteins involved in repair of DNA and maintenance of genome integrity. In normoxia, DNA damaged by radiotherapy is oxygen fixed and therefore permanent, producing predominantly double stranded DNA breaks. In hypoxia, the lack of oxygen results in only transient DNA damage. In addition, hypoxic conditions increase levels of replication stress. Activation of transducers of the DNA damage signal including DNA-PK, ATM, and ATR (also relevant for replication stress) have been reported. However, this is often independent of activation of sensing molecules including the MRN complex and Ku70/80, which have been shown to be downregulated. Likewise, effectors of DNA damage repair across multiple pathways have been shown to be downregulated. The results of these alterations are numerous, from resistance to cell killing by chemo/radiotherapeutics to genomic instability. Considerably more research is needed to elucidate the downstream mechanisms of these hypoxic alterations. RNR ribonucleotide reductase, MRN MRE11-RAD50-NBS1 complex, ATM ATR mutated, ATR ataxia telangiectasia Rad3 related, DNA-PKcs = DNA protein kinase catalytic subunit, LIGIV = DNA ligase 4, ATRIP = ATR-interacting protein, MutSα = complex of MSH2 and MSH6, MutLα = MLH1 and PMS2 complex. *W*ang^141^, Blackford^142^, Gaillard^143^ and Jiricny^144^.

The MRE11-Rad50-NBS1 (MRN) complex is responsible for sensing DNA damage and initiating both the HR and NHEJ pathways by recruitment of transducers such as ATM via NBS1^67^. While the MRN complex is considered the main sensor responsible for recruiting and activating ATM following damage, ATRIP (ATR-interacting protein) and Ku70/80 are sensors responsible for recruitment of ATR and DNA-PKcs respectively^67^ (Fig. 3), though there are many overlapping interactions. ATMIN (ATM-interacting protein), with roles in replication stress (RS), genome stability and the base excision repair (BER) pathway, has also been shown to recruit ATM independent of DNA damage^68^.

Repression of the MRN machinery has been seen in chronic hypoxia (>5 days) in a medulloblastoma model, with transcriptomic downregulation of both MRE11A and NBS1, resulting in downregulation of etoposide-induced ATM and p53^69^. NBS1 has also been shown to stabilise HIF-1α, particularly in response to IR^70^, while HIF-1α has been shown to downregulate NBS1. The authors of one study (where reduction of NBS1 was seen after 16 h in 1% O_2_) noted that this repression resulted in the induction of γH2AX and 53BP1 foci in hypoxia, suggesting the presence of DNA breaks^59^. Interestingly, all components of the MRN were found to be downregulated both at the mRNA and protein level in NSCLCs harbouring EGFR mutations incubated in severe hypoxia (0.1% O_2_). This downregulation in EGFR-mutated cells correlated with their increased sensitivity to EGFR-inhibiting drugs^71^.

Sensing of damaged DNA is a crucial step in the initiation of repair and begins with changes to the chromatin^72^. γH2AX, a phosphorylated variant of histone H2AX, is induced by MRN activation and accumulates at sites of damage in the chromatin, preceding recruitment and necessary for retention of key DDR signalling proteins including MRN and ATM^73^. Studies also show that γH2AX is crucial for retaining mediators such as 53BP1 (p53 binding protein 1), MDC1 (mediator of DNA damage checkpoint 1) and BRCA1 at sites of damage^66^. H2AX is primarily phosphorylated by ATM, but can also be phosphorylated by ATR and DNA-PKcs^63^. Indeed, as well as by radiation and chemotherapies, γH2AX has also been shown to be induced by hypoxia, following replication fork stalling. This phosphorylation has been shown in chronic, severe hypoxia to occur in a HIF, ATR or ATM-dependent manner^74–77^. Crucially, some evidence has shown the phosphorylation of H2AX present only in proliferating cells^77,78^. The downstream effects of this activation have been linked to other consequences of hypoxic regulation including angiongenesis^79^ via induction of VEGF^80^. Experimentally, resolution of γH2AX foci after irradiation is often used as a marker of DSB repair, as theory dictates that the phosphorylation should disappear after damage is repaired. However, in hypoxia this heavily relied upon protocol may necessitate further fine-tuning.

Ku70 and Ku80 (together forming a heterodimeric complex) tether damaged DNA at breaks and are key sensors of DSBs, responsible for recruitment of DNA-PKcs as part of the NHEJ pathway^63^. The Ku complex has been shown to be both upregulated and downregulated by hypoxia in different studies^81^. One study found downregulation of Ku80 after 6 h in mild hypoxia (2% O_2_)^82^. Another using severe hypoxia (<0.1% O_2_) found upregulation of Ku70/Ku80 in A431 cells, alongside many other members of the NHEJ pathway and proteins generally involved in metastatic progression^83^. A study in human and mouse hepatoma cells, found upregulation of the Ku heterodimer upon incubation in hypoxia (1% O_2_) or with hypoxia mimics, and downregulation associated with HIF-1β-deficient cells^84^. Alternative sub-pathways of NHEJ also exist, possibly as insurance for when classical NHEJ mediators are inoperative. However, the impact of hypoxia on these pathways has not been extensively studied.

### Transducers

ATM and ATR are two of the most important proteins involved in transduction of the DDR. As PIKK family members they phosphorylate a number of proteins involved in propagating the signal and repairing DNA, as part of both the HR and NHEJ pathways, as well as undergoing auto-phosphorylation to maintain the response until DNA is repaired.

Broadly speaking, ATM has been shown to be activated, particularly in acute hypoxia. As shown in the study by Bencokova et al., the pattern of activation in this context does not match RT-induced ATM activation, which follows MRN recruitment to DSBs^85^, as ATM phosphorylation does not correlate with the presence of DSBs, often reduced or absent in severe hypxoia^28^. The authors of this study emphasized that ATM activation was specific to the level of hypoxia (only phosphorylated at 0.02% O_2_) and HIF-independent since phosphorylation was maintained even in HIF-knockout cells. Activation of ATM was attributed to autophosphorylation, the result of which was an activation of targets (much like DNA damage-induced ATM activation), but dependent on the activity of cell cycle regulator MDC1^28^. This study did not analyse RR, but the results suggest that ATM activation by hypoxia is likely enacted as a means of halting the cell cycle in order to allow for DNA repair.

The pattern of ATM activation in hypoxia, however, is not clear-cut and may depend on cancer type. ATM can be regulated by a number of factors as part of the hypoxic response, including MRN or ATMIN, but also by post-translational and epigenetic factors^86^. One study found that ATM was downregulated along with HIF-1α by a microRNA, miR-18, resulting in radiosensitivity^87^.

The study by Hashimoto et al.^88^, showed ATM activation alongside activation of a number of other key DDR or cancer-related proteins including DNA-PKcs, Akt and EGFR and decreased expression of mTOR after 12/24 h at <0.05% O_2_, with the clearest phosphorylation of ATM seen at 24 h. The increased activation of Akt and EGFR is notable, as a relationship between EGFR activation of Akt has been suggested to be involved in DSB repair and/or regulation of cell death pathways in hypoxia leading to RR, via interaction with DNA-PKcs^89–91^.

ATR in normal circumstances is active mostly during single-strand break (SSB) repair and RS. It has also been found to be activated in acute hypoxia^92^, particularly as a consequence of hypoxia-induced RS^93^. Loss of ATR has been shown to result in more cell death after hypoxia/reoxygenation^92^. This result has led to the increased interest in the use of ATR-inhibitors to radiosensitise tumours. Indeed, compounds like VE-822^94^ or siRNA-mediated depletion of ATR^95^, have been shown to radiosensitise tumours with or without hypoxia as a consideration^96^.

RS is a feature commonly associated with hypoxia as a result of a depleted nucleotide pool and enzymes necessary for replication^75,97^. RS induced by severe hypoxia (0.01% O_2_) has also been seen to alter activation and expression of members of the Fanconi Anaemia (FA) pathways, also involved in DDR. In one report, FANCD2 and FANC1 showed activation in acute hypoxia, followed by a decrease in transcription after chronic incubation. This response was found to be ATR-dependent and suggested to contribute to genome instability^98^.

Targets of ATM and ATR, including Chk1 and Chk2 have also been shown to be activated in hypoxia, linking reports of cell cycle dysregulation commonly observed in hypoxia^32,76,99^. This is of particular importance as cell cycle control is intrinsically linked to DDR and RR. Pires et al., showed transient Chk1 activation in 0.02% hypoxia, reducing by 18 h and resulting in distinct changes to the cell cycle. This work built on previous studies^97^ which showed replication arrest in hypoxia as a result of dNTP depletion. The results demonstrated that between 6 and 12 h in hypoxia Chk1 became involved in replication restart in G1/G2-phase cells and p53-dependent apoptosis in S-phase cells following reoxygenation. After 18 h, replication did not resume after reoxygenation, due to disassembly of the replisome^32^. This demonstrates the existence of a critical window in which the fate of hypoxic cells is decided. What happens next, downstream of cell cycle checkpoints and DDR pathways, is crucial in mediating RR. Downregulation of Chk1 and its downstream targets such as CDC25 has also been seen^100,101^. However, these studies used 1% oxygen, which likely exemplifies the difference in cellular response between severe and mild hypoxia.

Another study in 2016 profiled a combination of positive and negative regulators of the G2/M checkpoint in hypoxic and irradiated cells. The results showed downregulation of most of these regulators (including CyclinB1, Plk1, and Chk2) and upregulation of a few (including CDK1 and p21) after incubation in severe chronic (0.2% for 72 h) or acute (<0.03% 20 h) hypoxia. The study also showed an RT-induced G2 arrest with cells incubated in these conditions^102^. However, activation of these proteins was not assessed, which may have been helpful for interpretation of these results. For example, increased phosphorylation of Chk2 may be associated with the observed total protein decrease. Another study in 0.1% O_2_ demonstrated maximum phosphorylation of Chk2 after 72 h, a result dependent on ATM, MLH1 (involved in MMR) and NBS1. This activation resulted in phosphorylation of p53 and cell cycle arrest^99^.

Mild hypoxia has also been shown to activate DNA-PKcs, a transducer of the NHEJ pathway. Like with ATM and ATR, this activation does not correspond to detectable damage or to recruitment of the XRCC4-LIGIV complex, as would usually occur following DNA-PKcs signal transduction in DDR. But, this has been shown to increase expression of HIF-1α and the subsequent transcription of GLUT1, indicating promotion of an adaptive response mediated by DNA-PKcs^103^. ATM, ATR and DNA-PKcs have all been shown to stabilise HIF, and affect accumulation and subsequent transcription of HIF-target genes^84,104,105^. DNA-PKcs and HIF-1α have also been shown to work in combination with MEK/ERK signalling to impart RR in glioblastoma cells, with a reduction in MEK/ERK leading to a reduction in HIF-1α accumulation and activity, and a downregulation of HIF-1α induced by inhibition of DNA-PKcs, leading to radiosensitisation^106^.

Drug compounds targeting DNA-PK have gained interest, with a number of studies showing potential as radiosensitising agents. KU57788 and IC87361 were used in one study to radiosensitise tumours and found to work particularly effectively under severe hypoxia or anoxia^107^. This study also tested inhibitors for PARP1, a mediator of multiple DDR pathways, and found that they were less effective than DNA-PK inhibitors. A crucial finding of this study was that efficacy of the inhibitors was not correlated to DNA-PK expression, but to the expression of a gene (SLFN11) involved in fork repair. When this gene was depleted, cells became highly radioresistant and unable to be sensitised by the inhibitors. Concurrent with the characteristic of HNSCCs infected with HPV to be more radiosensitive, SLFN11 was also upregulated in these cancers, according to data from The Cancer Genome Atlas (TCGA)^107^. Not only is this protein implicated in RS, but has also been shown to inhibit translation of ATR and ATM after DNA damage^108^, again demonstrating the circularity of DDR functions in hypoxia.

Another study investigated the efficacy of a bioreactive prodrug of the IC87361 compound mentioned above (SN38023), as a means of targeting DNA-PK in the most radioresistant tumours with minimal effect on normal tissues^109^.

A strong radiosensitization effect was seen with NSCLC cells incubated in hypoxia (1% O_2_) and treated with a DNA-PK-inhibitor (M-3814). In this study it was found that a combination of carbon ion irradiation (a form of radiation technology with a lower dependency on oxygen) and DNA-PK inhibitor was considerably more effective than either treatment alone, or carbon ions in combination with an ATM inhibitor. Interestingly, the effect of ATM inhibition was no different if the cells were normoxic or hypoxic. On the contrary, DNA-PK inhibition was considerably more effective in hypoxia^110^. Similar studies have also found HR to be reduced under chronic, severe hypoxia, where NHEJ pathways are activated^111^. These results could suggest that there may be a preference for NHEJ at this level of oxygen, at least in some cancer models. It could also be that the cell cycle stage in which the cells are treated or tested could have an impact on the results, as HR has limited activity outside G2. In addition, in vitro, response to radiation in combination with hypoxia may depend on the order in which cells are irradiated and incubated in hypoxia. One study showed that cells irradiated in hypoxia (0.1%) had increased survival and repair of DNA as shown by γH2AX foci. However, when these cells were incubated in hypoxia (1% O_2_) following radiation, no difference was seen in survival. Interestingly, each of the three cell lines tested then showed different responses when incubated in hypoxia prior to irradiation, with the response dependent on activity of ATM and DNA-PKcs^112^. This highlights the recurring issue with this area of study, in that even minor differences in experimental methodology, and particularly neglecting to report precise methodology, can considerably interfere with interpretation of results.

Another issue with many of these studies is that RR following these conditions is rarely measured. However, very few reliable techniques exist to measure RR. Clonogenic survival is the gold standard, along with measuring the presence of γH2AX and 53BP1 foci by immunofluorescence, and the comet assay to quantify active DNA damage. Yet, both γH2AX and 53BP1 can be activated by hypoxia-activated ATM and other factors, and their presence doesn’t necessarily correspond to DSBs or reduced survival^76^. The comet assay is problematic too, with a somewhat subjective interpretation of results and the fact that it can detect both DSBs and SSBs, the latter of which are commonly seen in hypoxia-induced RS. Therefore, the implications of these alterations in hypoxia are not yet defined. Perhaps, like we have done with hypoxia as a single parameter^18^, what the research community needs is a genomic or proteomic signature of hypoxic-radioresistance against which we can compare effects of various alterations.

### Effectors

Thus, it seems clear that transducers of DNA damage including ATM and ATR, and to an extent their targets Chk1/2 are activated by hypoxia. Since these factors are involved in the initial stages of the DDR, it may follow that the downstream effectors of repair are likewise upregulated. However, a general consensus from the literature suggests that effectors of DSB repair are reduced in hypoxia^32^. This means that the 3-act response so clearly defined in normoxia becomes uncoupled in hypoxia, with each set of mediators running according to their own programmes.

These alterations have been shown to occur either post-translationally or at the mRNA level as a result of altered behaviours of transcription factors like the E2F factors, c-Myc and SP1 as well as changes to chromatin structure and microRNAs^113–118^.

A number of studies from Robert Bristow and Peter Glazer’s groups in the 2000s, showed that chronic, severe hypoxia resulted in downregulation of HR effector proteins, including RAD51 and BRCA1, in a number of cancer models^32,111,119–121^. In vitro experiments by Chan and colleagues showed that 72 h in <0.2% O_2_ resulted in a decrease in HR capacity, leading to an increased sensitivity to DNA damage-inducing agents Mitomycin C and cisplatin^113^. In a study by Bindra et al., Rad51 and BRCA1 were found to be decreased in 0.01% O_2_ alongside small reductions in expression of RAD54B and CSB, with mild increases in ERCC1 and RAD51B. This was also found to correlate to increases in VEGF^111^, confirming hypoxia adaption. Quite contrary to this, a study by Kang et al.^122^ in 2006 showed that exogenously expressed BRCA1 interacted with HIF-1α in hypoxia (0.1% up to 24 h), leading to upregulation of VEGF. This study did not however look at BRCA1 expression on its own. Meng and colleagues additionally found increases in HR and NHEJ effectors after incubations for 48–72 h in 0.2% O_2_^119^.

It may be possible that a reduced DDR functionality in hypoxic cells could be exploited, by targeting other parts of the DDR pathway and exerting a synthetic lethal effect. This has been highly successful in the case of tumours with BRCA1/2 mutations treated with PARP inhibitors outside of the context of hypoxia. Indeed, one study, after confirming downregulation of RAD51 and BRCA1 by hypoxia, found that treatment with PARP inhibitors was more effective and radiosensitising in hypoxic cells^123^. However, this was found to be ineffective in a later study using cycling hypoxia^124^. Clinical trials for PARP inhibitors in highly hypoxic HNSCCs are ongoing^125^.

Aside from HR and NHEJ, DDR pathways including MMR, BER and nucleotide excision repair (NER) are also of particular relevance in the context of hypoxia with possible involvement in regulating HIF-target gene transcription^126,127^. Several reports have found a reduction in MMR genes, including downregulation of MLH1, MSH2 and MSH6 in both mild (1%) and severe (0.01%) hypoxia^116,127,128^. One study showed that downregulation of these genes led to decreased sensitivity to DNA damage inducers like Bleomycin or IR^129^, which appears to be in contrast to the downregulation of effectors of other pathways as mentioned above. Another study found that a subset of MMR-deficient colon cancer cells incubated at <0.1% O_2_ were enriched after a period of culturing, suggesting that these conditions selected for DDR-deficient cells. The resulting selected clones were MMR-deficient and significantly more drug-resistant^130^. Similarly, a study investigating BER in colorectal cancer cells, found downregulation of a number of BER repair genes after 72 h in 0.2% O2 and subsequent sensitivity to damaging agents like hydrogen peroxide^131^. It is possible that the results of downregulation of MMR or BER repair proteins may have different consequences, depending on the conditions and also on capabilities of other pathways within the same cell.

Exactly how these alterations result in the more radioresistant phenotype we see in hypoxic tumours is unknown. A major consequence of downregulation of DNA repair however, is genomic instability (GI)^129^. GI is associated with both tumour aggressivity and progression^132^, as well as RR^133,134^, though only a few studies have linked it to hypoxia-induced RR. The connection between GI and RR is mostly indirect, with GI thought to lead to the development of radioresistant clones following periods of chromosomal rearrangements^135,136^. A number of studies have shown development of GI in hypoxia^66^, a feature particularly relevant for tumours lacking functional p53 as is common in many hypoxic cancers, as apoptosis will be less likely to occur^137^. Reoxygenation was also found to induce GI after downregulation of MMR genes^58^. The discordance between activation of DDR transducers and downregulation of DDR effectors, as well as altered cell cycle regulation and upregulation of survival pathways in hypoxia has also been linked to GI^38^.

## Conclusions and future perspectives

Decades of research have allowed us to develop an understanding of how low oxygen conditions impact cancer cell survival. Though discrepancies in the literature are evident, partially as a result of incomprehensive reporting of hypoxic conditions, a picture of genetic reprogramming in the hypoxic tumour has developed. We see that hypoxic tumours exhibit high levels of RS and activation of DDR transducers ATM, ATR and DNA-PKcs independent of DSBs, and downregulation of DDR effectors. Alongside these changes we also see upregulation of HIF-target genes such as VEGF, altered cell cycle control and apoptosis, and GI. These factors together produce a more aggressive tumour able to overcome and resist cytotoxic effects of radiation.

There remain areas of research that urgently require more work. Research into the hypoxic control of the DDR has not thus far allowed us to modify treatment plans to improve therapy success rates. Perhaps the one exception being Nimorazole, a hypoxia-activated prodrug, now part of the standard of care in combination with radiotherapy for HNSCC patients in Denmark^15–17,24^ with trials ongoing in the UK^138^. Other developments, for example those that actively target the DDR such as DNA-PKcs-targeting prodrugs, ATR inhibitors or exploitation of GI, may allow us to more directly treat radioresistant hypoxic tumours. Indeed, even development of less oxygen-reliant radiotherapies such as carbon ion therapy, may help us overcome the RR induced by hypoxic tumours. However, until a full understanding of how RR is mediated by the alterations induced by hypoxia, meaningful clinical translations may not be possible.
